# The risk of jiggly fat in aging

**DOI:** 10.18632/aging.102147

**Published:** 2019-08-06

**Authors:** Patricia Corrales, Marina Martín-Taboada, Gema Medina-Gomez

**Affiliations:** 1 Department of Basic Sciences of Health, Area of Biochemistry and Molecular Biology, Universidad Rey Juan Carlos, Alcorcon-Madrid, Spain

**Keywords:** aging, adipose tissue, insulin resistance, caloric restriction

Aging is a multifactorial physiological process of decay associated with an increased risk of metabolic alterations, such as, obesity, insulin resistance (IR), and other manifestations associated to metabolic syndrome in both humans and rodents. Moreover, aging is characterized by an increased total adiposity and a redistribution of adipose tissue defined as loss of subcutaneous white adipose tissue (WAT) with expansion of visceral fat [1]. In fact, in advanced age the visceral fat seems to be the most affected tissue and responsible for the dysfunction of the other tissues [2,3]. Contrary to this, the subcutaneous adipose tissue, known as the “jiggly fat”, seems to be harmless and may even be a protective tool against some other age-related diseases. However, we have shown that at early stages of aging the subcutaneous adipose depot presented a lack of plasticity and functionality. Thus, the expansion of visceral adipose tissue might lead to peripheral lipotoxicity determined by a defect in the subcutaneous adipose tissue capacity to accumulate fat in the early stages of aging, at least in mice [4].

Changes in overall insulin sensitivity might reflect an alteration in insulin action during the aging process, but not all the insulin target tissues (adipose tissue, liver and muscle, mainly) are affected by age-related IR in the same way and at the same time. WAT shows a decreased glucose uptake in response to insulin action, which usually begins with the release into the circulation of free fatty acids and diacylglycerol [5]. When these lipids reach their target tissues, they could alter the insulin signaling pathway by inhibiting the insulin receptor substrate (IRS) as well as the phosphoinositol-3-kinase (PI3K). Another alteration caused by these lipids could be the prevention of the vesicles translocation from the cytoplasm to the cell membrane, which contains the glucose transporter (GLUT). Fatty acids can also induce the expression of proinflammatory cytokines and kinases such as TNFα or c-Jun kinases (JNK), which are known to cause inactivation or degradation of IRS [6]. Taken together, an excess of fatty acids associated to age could modify the glucose uptake by altering the pathway of protein kinase B (PKB or AKT), a key step in the physiological effects of insulin.

**Figure 1:**
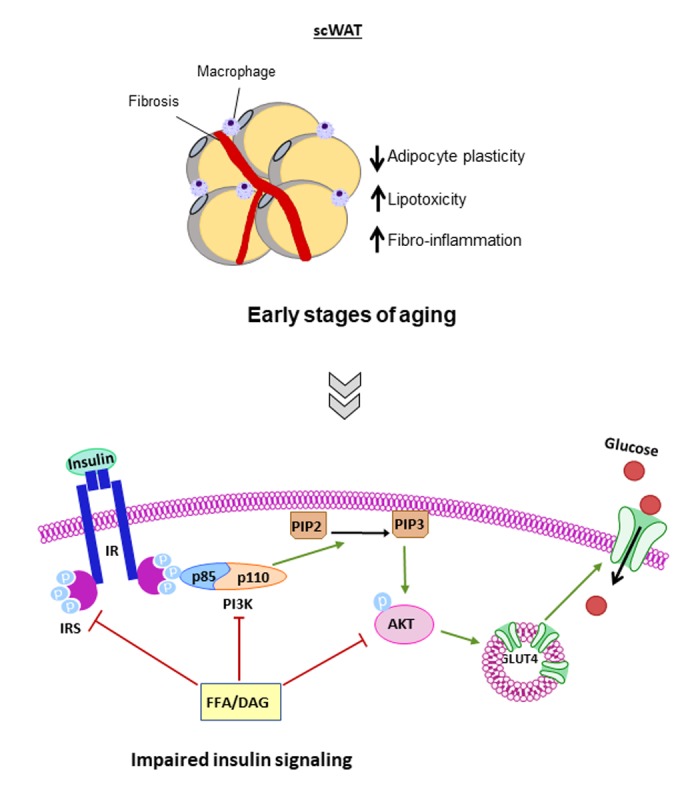
**Altered processes in Subcutaneous WAT during the first stages of aging**. Subcutaneous WAT (scWAT) shows a defect in hypertrophy and/or hyperplasia, fibrosis and inflammatory processes and insulin resistance (IR) appears progressively in this tissue during the first stages of aging. Insulin receptor substrate (IRS), phosphoinositol-3-kinase (PI3K), glucose transporter (GLUT), c-Jun kinases (JNK), protein kinase B (PKB or AKT), phosphatidylinositol (3,4,5)-tris- phosphate (PIP3), phosphatidylinositol 4,5-bisphosphate (PIP2), free fatty acids (FFA) and diacylglycerol (DAG).

Accretion of visceral fat, rather than subcutaneous fat, has been associated with IR development with aging in WAT [2]. We hypothesize that it is possible that impaired insulin response in subcutaneous white adipose depot could represent an early step leading to the overall IR observed at the first stages of aging, even before glucose intolerance develops. Subcutaneous WAT shows a defect in hypertrophy and/or hyperplasia, fibrosis and inflammatory processes during the first stages of aging (Figure 1). IR could appear progressively in this tissue, whereas in the muscle, the liver or, even at a low grade, in visceral fat a decrease of insulin sensitivity is not dilucidated until advanced aging [4].

One of the non-pharmacological approaches to delay the deleterious effects of age-related metabolic diseases is caloric restriction (CR). It has been shown that CR has physiological effects on lifespan and can reduce body weight with a decrease of adiposity [3,7]. The reduction of adiposity by CR leads to an improvement of the metabolic status and has multitude of beneficial effects against age-associated metabolic alterations such as IR [1,8]. However, the mechanisms involved in the amelioration of age-related effects, such as IR, by CR are not well understood.

In summary, evidence shows that subcutaneous WAT could be the first adipose depot to present age-related alterations (plasticity and functionality) because of the IR already present during early stages of aging. Moreover, the IR may be promoted early by an excess of fatty acids, which could be related to modifications in the glucose uptake as a consequence of alterations in the PI3K/AKT pathway. Furthermore, it will be interesting to elucidate the age-related alterations in the adipose tissue at early stages of aging and whether the subcutaneous adipose depot could be the main contributor to IR at this age. In addition, it is reasonable to hypothesize that a moderate long-term CR could be one of the best interventions to ameliorate these age-related alterations.
